# pH-Dependent Antimicrobial Properties of Copper Oxide Nanoparticles in *Staphylococcus aureus*

**DOI:** 10.3390/ijms18040793

**Published:** 2017-04-08

**Authors:** Yi-Huang Hsueh, Ping-Han Tsai, Kuen-Song Lin

**Affiliations:** 1 Graduate School of Biotechnology and Bioengineering, Yuan Ze University, Taoyuan 320, Taiwan; n8808701@gmail.com; 2 Department of Chemical Engineering and Materials Science, Yuan Ze University, Taoyuan 320, Taiwan; kslin@saturn.yzu.edu.tw

**Keywords:** copper oxide nanoparticles, *Staphylococcus aureus*, XANES, EXAFS

## Abstract

The antimicrobial properties of CuO nanoparticles have been investigated, but the underlying mechanisms of toxicity remain the subject of debate. Here, we show that CuO nanoparticles exhibit significant toxicity at pH 5 against four different *Staphylococcus aureus (S. aureus)* strains, including Newman, SA113, USA300, and ATCC6538. At this pH, but not at pH 6 and 7, 5 mM CuO nanoparticles effectively caused reduction of SA113 and Newman cells and caused at least 2 log reduction, whereas 20 mM killed most strains but not USA300. At 5 mM, the nanoparticles were also found to dramatically decrease reductase activity in SA113, Newman, and ATCC6538 cells, but not USA300 cells. In addition, analysis of X-ray absorption near-edge structure and extended X-ray absorption fine structure confirmed that *S. aureus* cells exposed to CuO nanoparticles contain CuO, indicating that Cu^2+^ ions released from nanoparticles penetrate bacterial cells and are subsequently oxidized intracellularly to CuO at mildly acidic pH. The CuO nanoparticles were more soluble at pH 5 than at pH 6 and 7. Taken together, the data conclusively show that the toxicity of CuO nanoparticles in mildly acidic pH is caused by Cu^2+^ release, and that USA300 is more resistant to CuO nanoparticles (NPs) than the other three strains.

## 1. Introduction

*Staphylococcus aureus (S. aureus)* causes skin and soft tissue infections, toxic shock syndrome [1,2], and is also the major cause of nosocomial [3] and community-acquired bacteremia [4]. The emergence of methicillin-resistant *S. aureus* (MRSA) has become a serious problem [5], against which only a few antibiotics are effective. In addition, many women worldwide suffer from vaginal infections such as bacterial vaginosis caused by *S. aureus* [6]. The normal vaginal pH is approximately 4 to 4.5. During menstruation, *Lactobacilli* spp. bacteria appear to be unable to maintain the vaginal pH, and the rise in pH corresponds to rises in *S. aureus* levels or infection by other pathogenic bacteria [6,7]. Studies have shown that menstrual toxic shock syndrome (mTSS) is associated with vaginal colonization of *S. aureus* [8]. Hence, new strategies are urgently needed to identify and develop next-generation antibiotics, especially since *S. aureus* strains evolve more quickly than antibiotics can be developed.

Metal nanoparticles show promise as a new class of antimicrobials or as an alternative to antibiotics, as these agents non-specifically target most bacteria and fungi, and biocidal agents based on highly ordered metal nanoparticles have been developed [9,10,11]. These nanoparticles (Ag, Cu, CuO, and Au) were found to be active against different microorganisms, including fungi, Gram-negative *Escherichia coli* [12,13,14] and Gram-positive *S. aureus* [14,15]. Of these nanoparticles, CuO has been widely used, not only as an antimicrobial agent, but also as an industrial material. Indeed, copper and complexes of copper have been used as fungicides, bactericides, and algaecides for many centuries [16]. Moreover, the antimicrobial properties of CuO nanoparticles have been evaluated in many species, including *Escherichia coli* (*E. coli*), *Bacillus subtilis* (*B. subtilis*), *Pseudomonas aeruginosa* (*P. aeruginosa*), and *S. aureus* [17,18,19]. CuO nanoparticles have been found to be bactericidal against such antibiotic-resistant microbes as *S. aureus* [18], but have limited cytotoxicity against mammalian cells.

It is believed that the toxicity of CuO nanoparticles is not solely due to the release of Cu^2+^ ions [20]. For example, bacterial contact with nanoparticles [21] was proposed to be essential for toxicity, as were size [19] and surface coatings [21]. Indeed, smaller CuO nanoparticles appear to be more toxic [22], although commercially available nanoparticles differ broadly in size, morphology, and degree of agglomeration. In particular, Azam et al. [23] found that small CuO nanoparticles around 20 nm have significantly stronger antibacterial activities against Gram-positive *B. subtilis* and *S. aureus*, and against Gram-negative *E. coli* and *P. aeruginosa*. On the other hand, Gilbertson et al. [21] found that CuO nanosheets (~250–1000 nm^2^ × 15 nm thick) have better antimicrobial activity against *E. coli* K12 than CuO nanopowders (<50 nm).

The mechanisms underlying CuO nanoparticle toxicity remain the subject of intense debate. The nanoparticles may release Cu^2+^ ions, and generate reactive oxygen species that induce oxidative stress. Indeed, Cu^2+^ ions released from nanoparticles were found to be a key determinant of toxicity [24,25,26]. Alternatively, CuO may interact with and penetrate bacterial membranes, as was observed by Gilbertson et al. [21] on transmission electron micrographs of *E. coli* exposed to CuO nanoparticles. We note, however, that it is also possible that released Cu^2+^ ions penetrate cells and are eventually oxidized to CuO.

In this study, we examined the activity of CuO nanoparticles against four different *S. aureus* strains including three methicillin-sensitive *S. aureus* (MSSA) strains (Newman, SA113, and ATCC6538), and one methicillin-resistant *S. aureus* (MRSA) strain (USA300), and assessed the mechanisms underlying toxicity via X-ray absorption spectroscopy, an excellent tool to determine valence and local structure.

## 2. Results

### 2.1. CuO Nanoparticles Inhibit Growth

**Figure 1:**
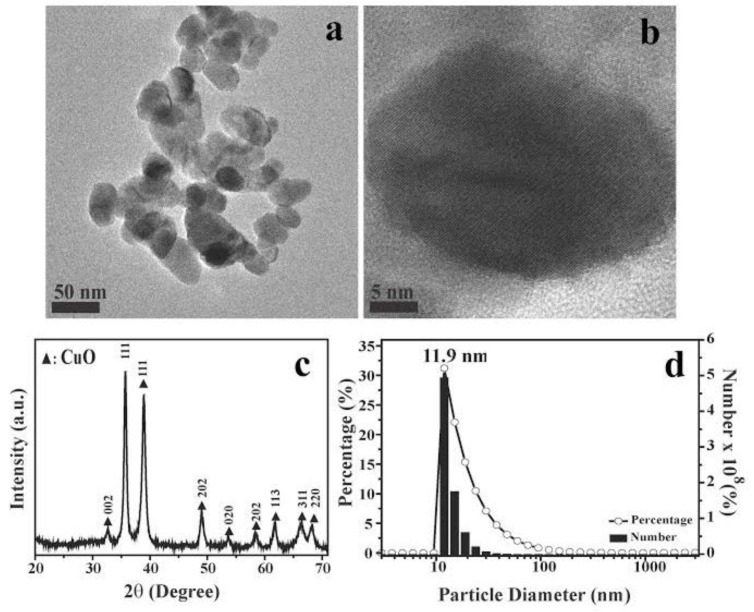
Shape, crystal structure, and particle size characterization of CuO nanoparticles (NPs). (**a**,**b**) scanning electron microscopy (SEM) images of CuO NPs used in this study; black bars: (**a**) 50 nm and (**b**) 5 nm; (**c**) X-ray diffraction (XRD) patterns of synthesized CuO NPs; (**d**) Size distribution histograms of CuO NPs. The data are representative of two separate experiments.

CuO nanoparticles vary broadly in terms of size and morphology [27], which are believed to be major determinants of toxicity. Hence, it is important to characterize the size and structure of nanoparticles used, in order to compare results across studies. In this study, we used CuO nanoparticles purchased from Alfa Aesar Corp. (Ward Hill, MA, USA), and which were found by field emission (FE)-scanning electron microscopy to be about 10 to 40 nm in average diameter (Figure 1a,b,d). X-ray diffraction patterns showed nine main characteristic diffraction peaks at [110], [111], [111], [202], [020], [202], [113], [311], and [220] (Figure 1c), which correspond to 2*θ* = 32.7°, 35.7°, 38.9°, 48.9°, 53.7°, 58.5°, 61.8°, 66.5°, and 68.3°, respectively, based on diffraction patterns for monoclinic CuO (JCPDS Card Number PDF#48-1548). The overall X-ray diffraction patterns were comparable with previously reported spectra [28,29,30].

**Figure 2:**
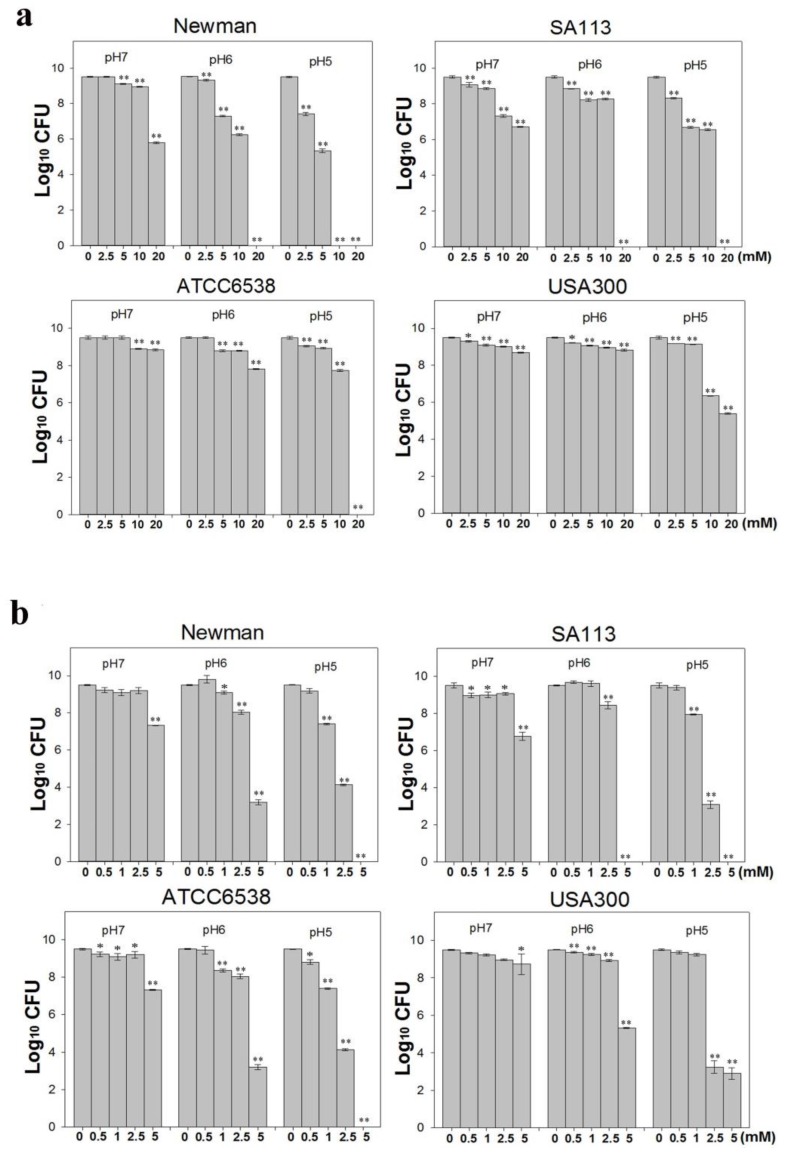
Effects of different CuO-NP concentrations on *S. aureus* antibacterial activity. Four *S. aureus* strains grown in Mueller Hinton broth (MHB) media were treated with different concentrations of (**a**) CuCl_2_ or (**b**) CuO NPs at 37 °C with shaking at 180 rpm for 24 h and evaluated by plating counts to determine antibacterial activity. The data are expressed as the means ± standard deviation (SD) of two separate experiments, with three replicates. ** indicates *p <* 0.01 and * indicates *p <* 0.05 as determined by one-way analysis of variance (ANOVA) relative to the control sample at different pH values.

*S. aureus* is one of the most concerning pathogens because of antibiotic resistance [31]. Therefore, we assessed the activity of CuO nanoparticles against four different *S. aureus* strains. Briefly, we treated ~1 × 10^7^ colony forming units (CFU)/mL freshly grown *S. aureus* cultures with 0–20 mM CuO nanoparticles, and evaluated bacterial growth over 24 h in Mueller Hinton broth (MHB) medium at pH 5, 6, and 7 (Figure 2a). Growth was not significantly inhibited at pH 7, although some inhibition was observed at concentrations over 10 mM. In particular, growth of the strains Newman and SA113 was reduced by three orders of magnitude in the presence of 20 mM CuO nanoparticles and at 10 mM, SA113 was reduced about two folds of magnitude (Figure 2b). In contrast, strains ATCC6538 and USA300 were more resistant, and were not significantly inhibited even at this concentration. At pH 6, growth of strains Newman and SA113 was reduced by 7 logs when exposed to 20 mM CuO nanoparticles. At 5 and 10 mM, growth was reduced by 2 and 3 log, respectively, in the Newman strain, and by one log in SA113. As observed at pH 7, strains ATCC6538 and USA300 were not significantly affected. At pH 5, growth was inhibited by around two orders of magnitude in strains Newman and SA113 treated with 2.5 mM nanoparticles (Figure 2b). At 5 mM, SA113 and Newman cells showed a reduction of approximately 3 log, and at 10 mM, Newman cells were all dead, and SA113 and USA300 cells showed a reduction of 2 log, whereas ATCC6538 cells had a reduction of one log. At a concentration of 20 mM, most strains were killed. Growth of strain USA300 was not significantly inhibited at CuO at concentrations lower than 5 mM, but was reduced by approximately one log at 10 mM. At 20 mM, growth of USA300 was inhibited by 4 logs, whereas ATCC6538 did not grow at all. Collectively, the data suggest that CuO nanoparticles were more toxic in mildly acidic conditions, and the USA300 strain was more resistant to the nanoparticles than the Newman, SA113, and ATCC6538 strains.

It is possible that in acidic conditions, CuO nanoparticles are more soluble, release copper ions more freely, and thereby attack *S. aureus* more effectively. Hence, we also tested 0–5 mM CuCl_2_, which releases copper ions rapidly and efficiently, against ~1 × 10^7^ CFU/mL *S. aureus* cultures in MHB media at pH 5, 6, and 7. At pH 7, CuCl_2_ did not significantly kill bacterial cells after 24 h (Figure 2b). Only at a higher concentration of 5 mM was growth reduced by 2 logs in Newman and SA113, and by one log in strain ATCC6538. At pH 6, strain SA113 was completely killed at 5 mM CuCl_2_, whereas the Newman and ATCC6538 strains showed a 6 log reduction and USA300 showed a 4 log reduction. At pH 5, all of the strains except USA300 were killed at concentrations higher than 5 mM CuCl_2_ (Figure 2b). In contrast, strain SA113 was inhibited by 6 logs at over 2.5 mM, by one log at 1 mM, and was killed at 5 mM. Similar results were obtained with the Newman and ATCC6538 strains. In contrast, strain USA300 was more resistant than other strains, was not inhibited at 1 mM, and was inhibited only by 6 logs at over 2.5 mM. Taken together, the data indicate that the strains are vulnerable to Cu^2+^ to various extents in acidic pH, and that the Newman, SA113, and ATCC6538 strains are the most sensitive, whereas the USA300 strain is the most resistant. Of note, we found that in suspensions of 20 mM CuO nanoparticles, 0.461, 0.398, and 0.265 mM Cu^2+^ was released at pH 5, 6, and 7, respectively (Table 1), suggesting that solubility increased with decreasing pH.

### 2.2. CuO Nanoparticles Alter the Redox Status in S. aureus

**Figure 3:**
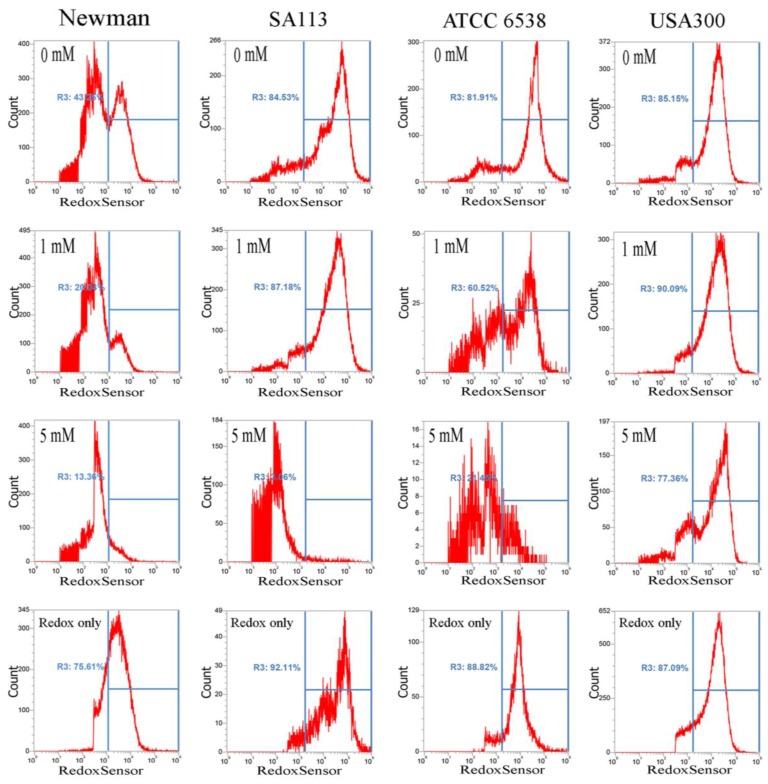

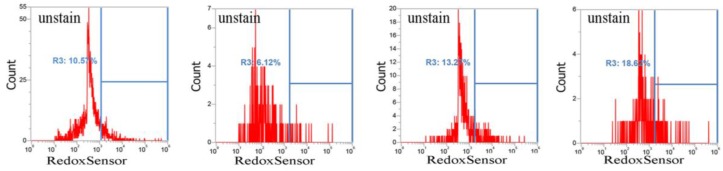
Analysis of RedoxSensor™ content in *S. aureus*. Four *S. aureus* strains were grown in MHB supplemented with CuO-NP concentrations of 0–5 mM for 3 h and the cells were then treated with RedoxSensor™ Green (green). The RedoxSensor™ fluorescence intensity is given in arbitrary units (a.u.) on the *X*-axis, and the *Y*-axis indicates cell counts as measured by flow cytometry. Phosphate buffer saline (PBS)-only and unstained cells were used as controls. RedoxSensor™ is fluorescent green color. The data are representative of two separate experiments.

It has been reported that CuO nanoparticles may increase levels of reactive oxygen species [17]. Thus, we examined whether these nanoparticles affect reductase activity in *S. aureus*. Briefly, overnight bacterial cell cultures, approximately 1 × 10^7^ CFU/mL, were treated with 0–5 mM CuO nanoparticles and washed after 3 h. Approximately 1 × 10^7^ CFUs were stained with RedoxSensor Green™ (Thermo Fisher Scientific, Eugene, OR,USA), and a green fluorescent dye was used to assess the oxidoreductase activity of four different *S. aureus* strains, and a red dye was used to assess membrane integrity. RedoxSensor staining in strains Newman, SA113, ATCC6538, and USA300 revealed that CuO nanoparticles generally reduced the geometric mean of reductase activity. For example, the geometric mean of reductase activity in the Newman, SA113, ATCC6538, and USA300 strains was 4079, 45,257, 39,085, and 17,140 arbitrary units (a.u.) for 0 mM CuO nanoparticles; 3151, 30,528, 16,353, and 20,342 a.u. at 1 mM; and 2378, 5778, 4822, and 22,665 a.u. at 5 mM, respectively. The percentage of gated cells in the total cell population was 43.25%, 84.53%, 81.91%, and 85.15% for 0 mM CuO nanoparticles; 20.04%, 87.18%, 60.52%, and 90.09% at 1 mM; and 13.36%, 3.06%, 21.45%, and 77.36% at 5 mM (Figure 3). As demonstrated by these data, reductase activity was significantly decreased by exposure to 5 mM CuO nanoparticles in the Newman, SA113, and ATCC6538 strains, but not in USA 300 cells (Figure 3).

**Figure 4:**
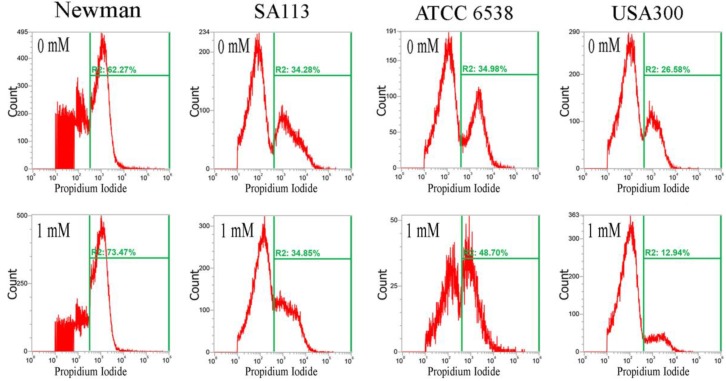

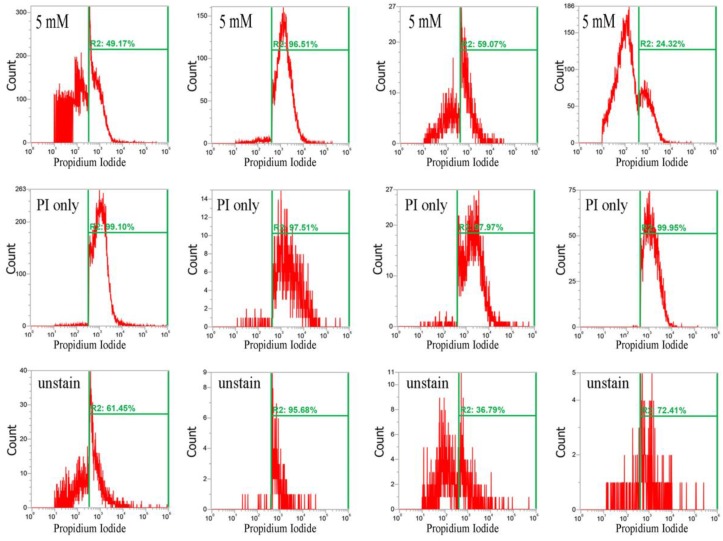
Analysis of propidium iodide (PI) staining in *S. aureus*. Four *S. aureus* strains were grown in MHB supplemented with CuO-NP concentrations of 0–5 mM for 3 h and the cells were then subjected to PI (red) staining. The PI fluorescence intensity is given in arbitrary units (a.u.) on the *X*-axis, and the *Y*-axis indicates cell counts as measured by flow cytometry. PBS buffer-only and unstained cells were used as controls. PI staining is fluorescent red color. The data are representative of two separate experiments.

Furthermore, CuO nanoparticles severely compromised the integrity of cell membranes after just 3 h, and dramatically increased the percentage of cells stained with propidium iodide. In the Newman, SA113, ATCC6538, and USA300 strains, the geometric mean of the propidium iodide (PI) staining was 1116, 1771, 2210, and 1166 a.u. at 0 mM CuO nanoparticles; 1052, 1641, 1119, and 1808 a.u. at 1 mM; and 695, 1464, 766, and 961 a.u. at 5 mM, respectively. The percentage of gated cells was 62.27%, 34.28%, 34.98%, and 26.58% at 0 mM; 73.47%, 34.85%, 48.70%, and 12.94% at 1 mM; and 49.17%, 96.51%, 59.07%, and 24.32% at 5 mM, respectively (Figure 4).

The decrease in the percentage of gated Newman cells positive for PI staining under treatment with 5 mM CuO may have still been representative of cell death, as PI is not detectable when cells are completely dead and the membrane has disintegrated. Additionally, USA300 cells showed a slight decrease in viability at 1 mM but no decrease at 5 mM. Collectively, the results show that CuO nanoparticles significantly decrease reductase activity but significantly increase membrane permeability in the Newman, SA113, and ATCC6538 strains, but not in USA300 cells (Figure 4).

### 2.3. Analysis of CuO Nanoparticle Fine Structure in S. aureus Cells

**Figure 5:**
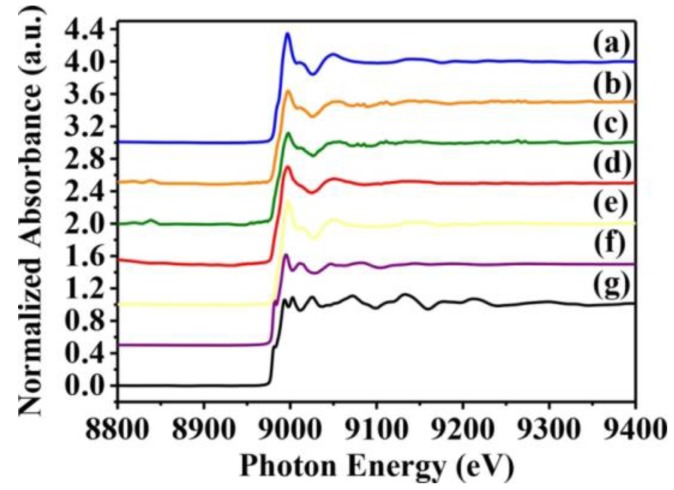
Normalized *K*-edge XANES spectra of CuO standards and *S. aureus* cells treated with CuO NPs. *K*-edge Normalized XANES spectra of (**a**) SA113; (**b**) ATCC6538; (**c**) USA300; (**d**) Newman; (**e**) CuO; (**f**) Cu_2_O; and (**g**) Cu. *S. aureus* cells were treated with 10 mM CuO NPs. The data are representative of two separate experiments.

Whether toxicity is due to the CuO nanoparticles themselves or to Cu^2+^ ions released from them is yet to be resolved. Hence, we used X-ray absorption near-edge and fine structure spectroscopy to characterize the oxidation state and fine structure of CuO atoms in *S. aureus* cells. Near-edge structure spectra can provide information on valence states, and can be compared to reference standards to determine oxidation state. Thus, we collected normalized copper K-edge spectra from Newman and SA113 strains exposed to 10 mM CuO nanoparticles, and from ATCC6538 and USA300 cells exposed to 10 mM CuO nanoparticles. Spectra from CuO, Cu_2_O, and Cu standards are presented in Figure 5e–g, while derivative spectra are shown in Figure 5. At around 9000 and 9015 eV, spectra derived from *S. aureus* cells treated with CuO nanoparticles overlapped with those of CuO and Cu_2_O standards, which contain several sharp absorption peaks between 9000 and 9050 eV. However, peaks at 9050 eV were consistent only with CuO (Figure 5e), indicating that cells likely contain CuO (Figure 5a–d).

On the other hand, X-ray absorption fine structure spectra are element-specific and highly sensitive, and therefore are used to determine the chemical state of target species present at very low concentrations. Thus, we used fine structure spectra to analyze atomic neighbors, bond length, and coordination number of CuO and CuS in *S. aureus* cells exposed to different CuO nanoparticles. Figures S1–S4 show the k^3^-weighted and least square-fitted CuO K-edge fine structure inverse Fourier transform oscillation spectra from CuO and CuS standards, and from *S. aureus* cells treated with CuO nanoparticles. Electronic scatter between CuO and CuS in the first electron shell was assessed on inverse Fourier transform spectra between 1 and 2 Å, and results show (Figures S1–S4a,b) that CuO, but not CuS, closely approximates the spectra derived from *S. aureus* cells exposed to CuO nanoparticles. Further analysis revealed that the bond distance spectra of nanoparticle-treated *S. aureus* cells clearly overlapped with that of CuO at 1.50 Å (Figures S1–S4a,b), thus confirming the coordination shell as Cu(I)-O. To further differentiate between the fitting for CuO and that for CuS, we quantitatively analyzed the bond length and coordination number of CuO and CuS in *S. aureus* cells treated with CuO nanoparticles. For Cu-O, the bond length was 1.95, 1.97, 1.97, and 1.97 Å in Newman, SA113, USA300, and ATCC6538 cells, with coordination numbers of 2.83, 2.41, 1.96, and 1.74. For CuS, the bond length was 2.11, 1.31, 2.13, and 2.13 Å in Newman, SA113, USA300, and ATCC6538 cells, with coordination numbers of 4.10, 3.00, 2.61, and 2.34.

*R*-factor analysis further confirmed the goodness of model fit (CuO < CuS, with lower *R*-factors indicating tighter and better fits in Table 2). All Newman, SA113, USA300, and ATCC6538 cells had a smaller *R*-factor with CuO than with CuS. This indicates that CuO had a better fit than CuS. Collectively, the data indicate that the microbial toxicity of CuO nanoparticles is likely due to released Cu^2+^ ions that penetrate *S. aureus* cells to cause toxicity, and is subsequently oxidized intracellularly to CuO.

## 3. Discussion

In this study, we found that CuO nanoparticles at the concentration of 20 mM (1600 ppm) kill *S. aureus* Newman, SA113, and ATCC5638 cells but kill USA300 cells to a lower degree in MHB medium at pH 5 (Figure 2b). However, at pH 6 and 7, all four *S. aureus* strains were more resistant to CuO nanoparticles. Of note, we also found that CuO nanoparticles were more soluble at pH 5 than at pH 6 and 7, suggesting higher toxicity of the nanoparticles at lower pH owing to the increased release of Cu^2+^ ions. Accordingly, all four *S. aureus* strains were killed more effectively at much lower concentrations of CuCl_2_ at all pH values, indicating that Cu^2+^ ions are key agents of toxicity. However, acidic pH may also enhance the ability of CuO nanoparticles to penetrate cells.

Previously, Kaweeteerawat et al. [22] found that 20–100-nm CuO nanoparticles are more toxic than microsized CuO to Gram-negative *E. coli* and Gram-positive *Lactobacillus brevis*, with IC_50_ (half maximal inhibitory concentration) of 160 ppm and 3.6 ppm, respectively. In contrast, Azam et al. [23]. found that CuO nanoparticles sized approximately 29 nm are more effective against Gram-positive bacteria such as *S. aureus* (22 ppm) and *B. subtilis* (20 ppm) than against Gram-negative bacteria such as *E. coli* (25 ppm) and *P. aeruginosa* (28 ppm). However, Azam et al. [23] used the inhibition zone assay on agar plates, which is not ideal to test the minimum bactericidal concentration because bacterial cells cannot be counted and the cells were not grown on solid plates but were still alive. Thus, this method is not useful to measure cell death. We therefore used the minimum bactericidal concentration method to test the viability of *S. aureus* cells in MHB medium on treatment with different concentrations of CuO NPs and counted cell numbers by plating the cells. We found that the bactericidal concentration for CuO nanoparticles was approximately 1500 ppm for *S. aureus* SA113, Newman, and ATCC6538 strains at pH 5; this difference in resistance probably resulted from biological differences among strains in the present study, as is observed between methicillin-resistant and methicillin-sensitive *S. aureus*. Furthermore, our data suggest that the resistance of SA113, Newman, and ATCC6538 strains to CuO nanoparticles is comparable, as is that of strain USA300, an MRSA strain. Collectively, our data suggest that CuO nanoparticle toxicity depends on pH values and that different bacteria have varying sensitivities to nanoparticles.

To elucidate the mechanisms by which CuO nanoparticles kill bacterial cells, we stained cells with propidium iodide to test whether nanoparticles disrupt bacterial cell membranes in *S. aureus*. The concentration-dependent increase in membrane damage that we observed implies that nanoparticles increase membrane permeability, and possibly penetrate cells to cause protein toxicity. The observed decrease in reductase activity after exposure to nanoparticles suggests that increased levels of reactive oxygen species contribute to the loss of membrane integrity and cell viability. We note, however, that Li et al. [32] found that CuO nanoparticles do not generate reactive oxygen species even after 48 h of UV irradiation, indicating that the antimicrobial activity is not due to photocytotoxicity. However, they also found that ionization of CuO nanoparticles to Cu^2+^ ions might cause toxicity against *E. coli*. Indeed, whether toxicity is inherent to CuO nanoparticles or is dependent on Cu^2+^ ions that leach from nanoparticles is an ongoing debate. To clarify this issue, we assessed the fine structure of CuO nanoparticles internalized into *S. aureus* and found that CuO best fit the spectra obtained from cells exposed to nanoparticles (Figure 5 and Table 2).

Importantly, we now clearly demonstrate that toxicity depends on pH, possibly because of the increased solubility of CuO nanoparticles in acidic pH (Table 1). Consequently, the enhanced release of Cu^2+^ in acidic pH may elicit the production of reactive oxygen species and alter redox status. Upon internalization, Cu^2+^ ions may then be subsequently oxidized to CuO. This process might explain the decrease in reductase activity in four different *S. aureus* strains and increase in propidium iodide staining in the SA113 and ATCC5638 strains, even though the percentage of propidium iodide-gated cells increased. However, the increase in the percentage of gated Newman cells on PI staining with 5 mM CuO NPs may have been caused by cell death because PI was not detectable.

The XANES/EXAFS data show that CuO was present in bacterial cells cultivated with CuO NPs. It is possible that Cu^2+^ ions entered *S. aureus* cells to induce an reactive oxygen species (ROS) response, resulting in decreased RedoxSensor activity, but were subsequently deionized to CuO. This might explain the results observed in cultures cultivated with ≥5 mM CuO NPs, where RedoxSensor activity was significantly decreased but membrane permeability was significantly increased in the Newman, SA113, and ATCC6538 strains, but not in USA300 cells (Figure 3 and Figure 4). To our knowledge, we are the first to directly analyze CuO molecules internalized in bacterial cells by X-ray absorption near-edge and fine structure spectroscopy, and to demonstrate that Cu^2+^ ions are primarily responsible for toxicity.

In summary, our results indicate that CuO nanoparticle toxicity results from the release of Cu^2+^ ions that subsequently penetrate bacterial cells at lower pH. Therefore, CuO NPs can be used as therapeutic agents in bacterial vaginosis, where normal vaginal pH is 4–4.5. We are the first to demonstrate this toxicity against *S. aureus*, and our data highlight the bactericidal potential of engineered nanoparticles against such pathogens.

## 4. Materials and Methods

### 4.1. CuO-NP Characterization

CuO nanoparticles were purchased from Alfa Aesar Corporation (Ward Hill, MA, USA) and copper chloride (AL222011-250 g) was purchased from Sigma-Aldrich (St. Louis, MO, USA). Field emission scanning electron microscopy (FE-SEM) was performed using a Rigaku RU-H3R field emission scanning electron microscope (Rigaku, Tokyo, Japan) to characterize the prepared samples in terms of their morphology, structure and elemental composition. X-ray diffraction (XRD) was measured at 20°–80° (2θ) and a scan rate of 4° (2θ)/min on a Rigaku RU-H3R diffractometer, and was used to characterize the crystallity and solid phase structure of CuO NPs using monochromatic Cu K_α_ radiation at 1.5405 Å, 30 kV, and 20 mA.

### 4.2. Assessment of CuO-NP Antibacterial Effects

*S. aureus* USA300, SA113, Newman, and ATCC6538 strains were maintained in Mueller Hinton broth [33] (MHB) broth or on 1.5% Bacto agar plates at 37 °C. Overnight cultures of approximately 1 × 10^9^ CFU/mL of *S. aureus* cells were diluted 100-fold into 50 mL of MHB in 250 mL Pyrex flasks at pH values of 5, 6, and 7. CuO NPs were added to achieve final concentrations of 0, 2.5, 5, 10, or 20 mM. Cells were then grown for 24 h at 37 °C and agitated at 175 rpm in a rotary shaker. All cultures were serially diluted, plated on Luria-Bertani (LB) agar plates, incubated overnight at 37 °C, and then subjected to a colony count. All experiments were performed in duplicate and each value represents the mean of three technical replicates. Statistical significance was determined using a one-way analysis of variance (ANOVA).

### 4.3. RedoxSensor Measurement

Reductase activities of *S. aureus* strains were determined using a BacLight™ RedoxSensor™ Green Vitality Kit (ThermoFisher, Waltham, MA, USA) [34]. Overnight cultures of approximately 1 × 10^7^ CFU/mL in 50 mL of MHB at pH 5 were supplemented with the indicated concentrations of CuO NPs for 3 h at 175 rpm and 37 °C. Cells were washed with 1× PBS buffer twice, diluted 10-fold with the same buffer, and eventually mixed well with 1 μL of RedoxSensor™ Green reagent. In additional, 1 μL of propidium iodide (PI) was added to the mixture and incubated in the dark at room temperature for 5 min for assessment of cell membrane integrity. Stained cells (1 mL) in PBS were assayed by flow cytometry using an *Attune*™ *NxT* Flow *Cytometer* (ThermoFisher, Waltham, MA, USA). Fluorescence filters and detectors were all standardized with green fluorescence collected in the FL1 channel (530 ± 15 nm) and red fluorescence collected in the FL3 channel (>650 nm). Data were analyzed using *Attune*™ *NxT* Flow *Cytometer software*. PBS buffer only: only adding PBS buffer but not cells and fluorescent dyes. Unstained: bacterial cells did not stain with fluorescent dyes. PI only: bacterial cells stained with PI dye only without Redox dye. Redox only: bacterial cells stained with Redox dye but not PI dye. An amount of 0–5 mM of CuO NPs: Bacterial cells stained with both PI and Redox dyes at 0–5 mM CuO NPs. All parameters were collected as logarithmic signals. All experiments were performed in two separate experiments and data are representative of results from two separate experiments.

### 4.4. XANES and EXAFS Analyses

The XANES and EXAFS results were collected at the Wiggler beamline 17A1 at the National Synchrotron Radiation Research Center of Taiwan. The electron storage ring was operated at an energy level of 1.5 GeV with a ring current of 100–200 mA. A Si (1 1 1) double-crystal monochromator was used with an energy of 6–33 keV (BL17C1) and a resolving power (*E*/Δ*E*) of up to 7000. Data were recorded using a fluorescence or transmission Lytle ionization detector [35]. Photon energy was calibrated against the absorption edges of Cu atoms in CuO (8979 eV) *K*-edge experiments. Non-linear least-square fitting was used to generate structural parameter data such as bond length (R), coordination number (CN), and Debye–Waller factor (σ) for different coordination shells surrounding the absorbing atoms. Least-square algorithms were used to fit a straight line to the raw absorption data in the range of 50–200 eV below the edge position, and the XANES region was within 50 eV above the absorption edge. The spectra were recorded in digital format with a step size equivalent to less than 0.5 eV in the near-edge and a count time weighted to be proportional to *k*^3^ at high energy.

The program Athena (VI) with a linear pre-edge and polynomial post-edge background subtracted from the raw ln (*I*_t_*/I*_0_) data was used to normalize the data. The data were then analyzed using the Artemis (VI) software FEFF-8 [36,37,38]. The linear pre-edge region and a polynomial for the post-edge region were used to background-correct the samples after calibration, with later normalization. The normalized EXAFS spectra were then converted to wavevector *K* space and weighted by *K*^3^ to enhance dampened scattering oscillations. The data were then Fourier-transformed to yield the radial structure function [37]. These data directly reflect the average local regions around the absorption atoms. The software package IFEFFIT [36,38] was used to analyze spectral data.

The first coordination shell of Cu-Cu, Cu-O, and Cu-S for Cu, CuO, and CuS species was fitting with the experimental data calculated by the FEFF-8 program based on the crystallographic data of individual species. Variable parameters such as coordination number, interatomic distance, Debye–Waller factor, and inner potential correction were used to fit procedures. In the case of CuO *K*-edge XANES spectra, the intensity of the pre-edge absorption at 8979 eV was used to distinguish the oxidation states of copper atoms in Cu or CuO species in the different samples. All experiments were performed in two separate experiments and data are representative of results from two separate experiments.

### 4.5. Measurement of Soluble Copper

Copper cations are obtained by dissolving copper salts, such as copper nitrates, in pure water. Subsequently, the soluble copper species in the aqueous solution were collected from centrifugation and filtration through cellulose membranes (0.22 µm) (Millipore, Bedford, MA, USA) processes and then the accurate amounts were estimated by following methods previously described by Gunman et al. [39,40,41]. In this study, Cu cations freed from CuO NPs in MHB of various pH values (pH 5, 6, and 7) were evaluated; CuO NPs were added to 50 mL of MHB until the copper concentration reached 20 mM. The mixtures were centrifuged at 10,000× *g* (Centrifuge 5810R; Eppendorf, Hamburg, Germany) for 30 min and then filtered with cellulose membranes. The trapped nanoparticles on the membranes were then washed three times to remove the remaining Cu cations completely. Atomic absorption spectrometry (AAS) (Avanta M; GBS Scientific Equipment, Ltd., Braeside, Victoria, Australia) was eventually used to determine the concentration of copper solutions. All experiments were performed in duplicate, and each value represents the mean of three technical replicates.

## Figures and Tables

**Table 1:** Soluble copper released from CuO NPs in Mueller Hinton broth (MHB) medium at different pH values after 12 h of incubation at 37 °C. The data are representative of two separate experiments.

Sample Name	pH = 5	pH = 6	pH = 7
Average Concentration (mg/L)	36.64	31.60	21.08
Standard Deviation, SD (mg/L)	0.20	0.04	0.16
Average Concentration (mM)	0.461	0.398	0.265
Standard Deviation, SD (mM)	2.5 × 10^−4^	5.0 × 10^−7^	2.0 × 10^−3^

**Table 2:** Fine structural parameters of copper samples analyzed using EXAFS spectra. The data are representative of two separate experiments.

Sample	Shell (1st)	CN ^a^ (±0.05)	*R* (Å) ^b^ (±0.01 Å)	^c^Δ σ^2^(Å^2^)	*R*-Factor
Newman	Cu-O	2.83	1.95	0.00183	0.00355
	Cu-S	4.10	2.11	0.00983	0.05399
USA300	Cu-O	2.41	1.97	0.00659	0.00719
	Cu-S	3.00	1.31	0.01731	0.30124
SA113	Cu-O	1.96	1.97	0.00065	0.03782
	Cu-S	2.61	2.13	0.00725	0.04089
ATCC6538	Cu-O	1.74	1.97	0.0051	0.02559
	Cu-S	2.34	2.13	0.00755	0.06644

CN ^a^: Coordination number; *R* ^b^: Bond distance; σ ^c^: Debye–Waller factor.

## Supplementary Materials

Supplementary materials can be found at http://www.mdpi.com/1422-0067/18/4/793/s1.
