# Supplementary material for: pH-Dependent Antimicrobial Properties of Copper Oxide Nanoparticles in Staphylococcus aureus
Source: Int J Mol Sci. 2017 Apr 8;18(4):793. doi: 10.3390/ijms18040793 (PMC5412377; doi:10.3390/ijms18040793)
Supplement: Supplementary file 1 [file ijms-18-00793-s001.pdf]

pH-Dependent Antimicrobial Properties of Copper Oxide Nanoparticles in *Staphylococcus aureus*

Yi-Huang Hsueh[1,*] Ping-Han Tsai[1,] Kuen-Song Lin[2]

[1] Graduate School of Biotechnology and Bioengineering, Yuan Ze University, Taoyuan 320, Taiwan
[2] Department of Chemical Engineering and Materials Science, Yuan Ze University, Taoyuan 320, Taiwan
* Correspondence: yihhsueh@saturn.yzu.edu.tw (YHH); Tel.: +886-3-463-8800 (ext. 2410)

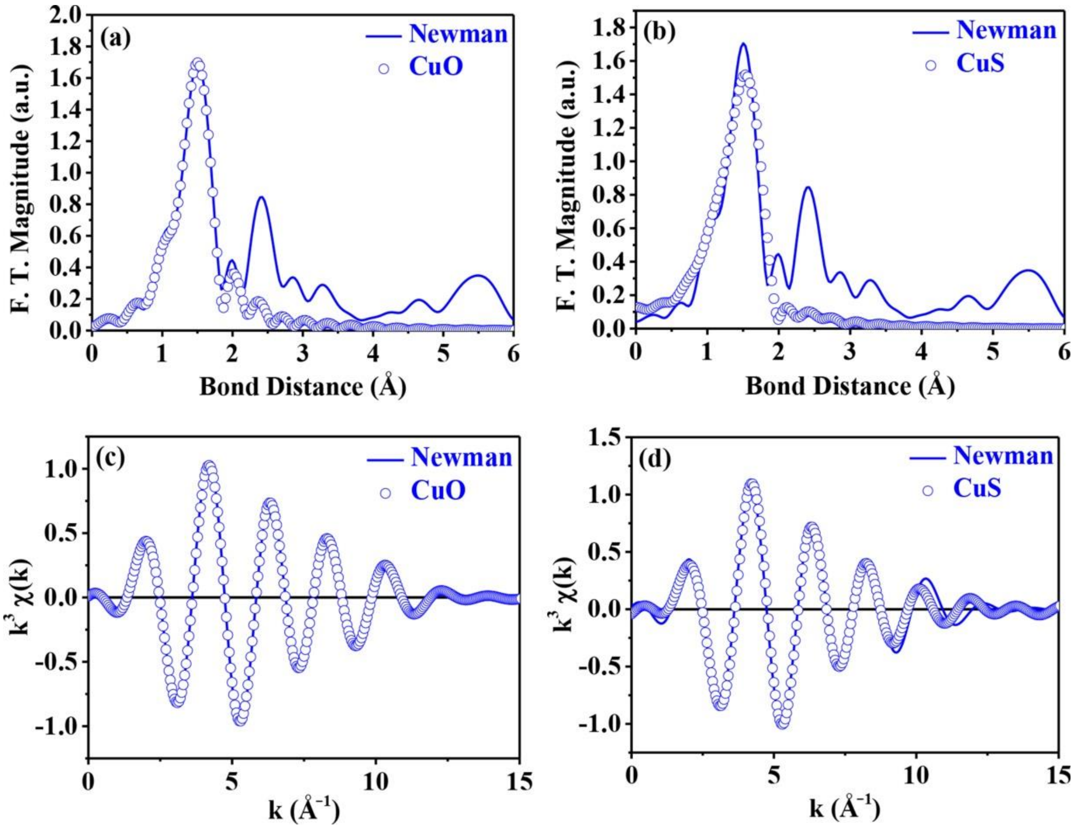

Figure S1. *K*-edge EXAFS oscillation $k^3\chi(k)$ of *S. aureus* Newman treated with 10 mM CuO NPs and fitted with (a), (c) CuO and (b),(d) CuS models. All experiments were performed in two separate experiments and data are representative of results from two separate experiments.

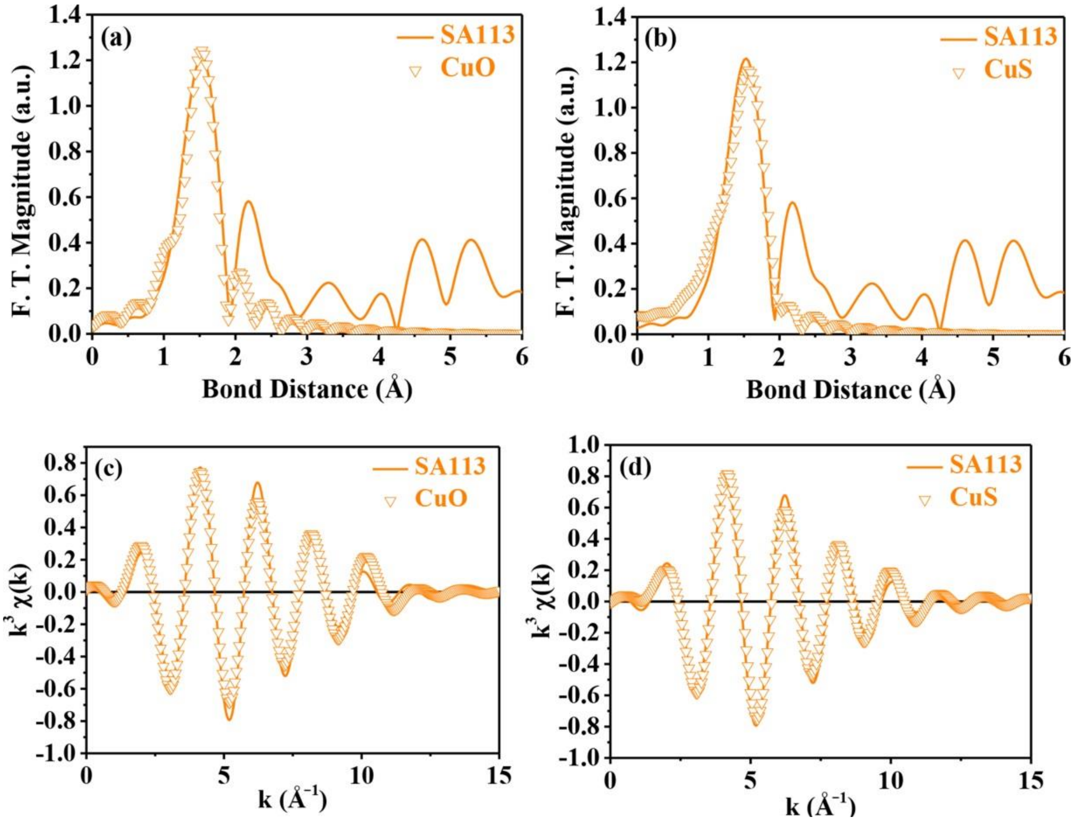

Figure S2. *K*-edge EXAFS oscillation $k^3\chi(k)$ of *S. aureus* SA113 treated with 10 mM CuO NPs and fitted with (a), (c) CuO and (b),(d) CuS models. All experiments were performed in two separate experiments and data are representative of results from two separate experiments.

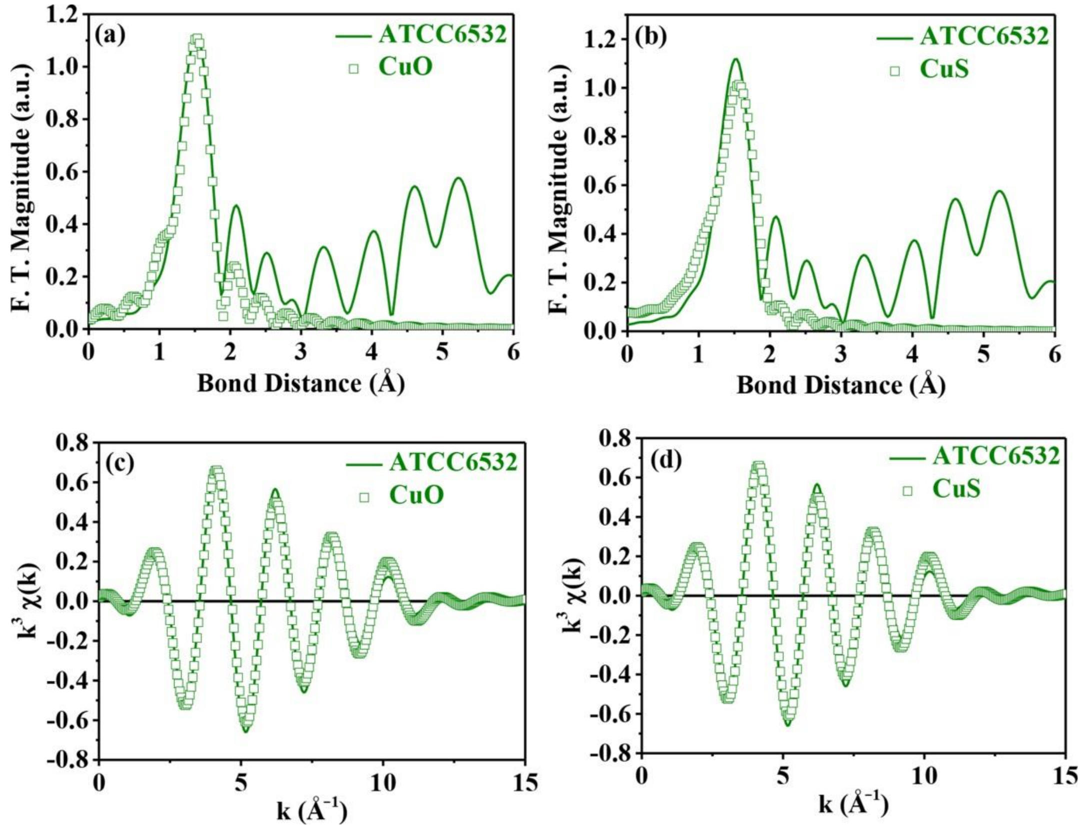

Figure S3. *K*-edge EXAFS oscillation $k^3\chi(k)$ of *S. aureus* ATCC6538 treated with 10 mM CuO NPs and fitted with (a), (c) CuO and (b),(d) CuS models. All experiments were performed in two separate experiments and data are representative of results from two separate experiments.

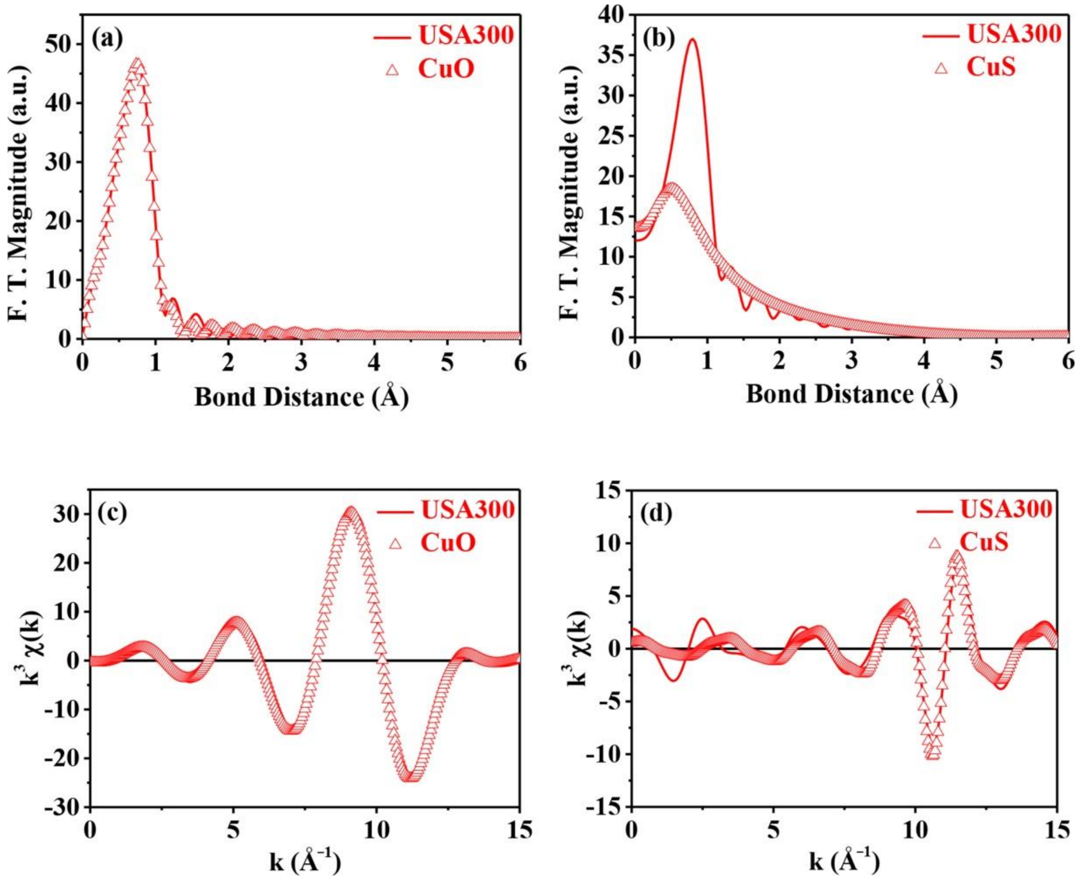

Figure S4. *K*-edge EXAFS oscillation $k^3\chi(k)$ of *S. aureus* USA300 treated with 10 mM CuO NPs and fitted with (a), (c) CuO and (b),(d) CuS models. All experiments were performed in two separate experiments and data are representative of results from two separate experiments.
